# Comparison of Antioxidant Capacity by Cooking Method of Apios (*Apios americana* Medicus) and Its Applicability as an Elder‐Friendly Food

**DOI:** 10.1002/fsn3.70102

**Published:** 2025-03-24

**Authors:** Dah‐Sol Kim, Ju Hong Park

**Affiliations:** ^1^ Graduate School of Convergence Science and Technology Pohang University of Science and Technology (POSTECH) Pohang Republic of Korea; ^2^ Department of Convergence IT Engineering Pohang University of Science and Technology (POSTECH) Pohang Republic of Korea

**Keywords:** 3D‐printed food, antioxidant, Apios, elder‐friendly food, hardness

## Abstract

Considering the deteriorated chewing ability among the elderly people, this study aimed to develop elder‐friendly foods that comply with the hardness standards set by Korean industrial standards (KS). First, we sought to establish an optimal cooking method by analyzing the antioxidant activity of Apios. Second, a gelling agent was incorporated into Apios cooked using the optimal method (sous‐vide) to make a mousse that meets KS hardness requirements by controlling its rheological properties. Third, we aimed to develop an elder‐friendly sous‐vide Apios that considers gastronomic characteristics by utilizing 3D printing technology. As a result, the loss rate of antioxidant capacity in the sous‐vide Apios was the lowest. A gelling agent was added to the sous‐vide Apios to achieve the desired hardness for KS, leading to the derivation of the regression equations: “*Y* = 1837.2*X* + 2829.2” for gelatin and “*Y* = 2937.1*X* + 1445.2” for agar. After preparing a mousse‐type sous‐vide Apios with KS's second‐level hardness based on these regression equations, optimal printing conditions were established to ensure stable output from the 3D printer. Considering these results, it is believed that the development of 3D‐printed elder‐friendly foods, which control rheological properties for easier chewing, will meet the diverse needs of elderly consumers. Furthermore, this innovation is expected to contribute to the growth of the elder‐friendly food industry by utilizing domestic agricultural resources such as Apios.

## Graphical abstract

As a result, the loss rate of antioxidant capacity in the sous‐vided Apios was the lowest. A gelling agent was added to the sous‐vide Apios to achieve the desired hardness for KS, leading to the derivation of the regression equations: “*Y* = 1837.2*X* + 2829.2” for gelatin and “*Y* = 2937.1*X* + 1445.2” for agar. After preparing a mousse‐type sous‐vide Apios with KS's second‐level hardness based on these regression equations, optimal printing conditions were established to ensure stable output from the 3D printer. A figure that best represents the scope of the paper. The image supplied should fit within the dimensions of 50 mm × 60 mm and be fully legible at this size.

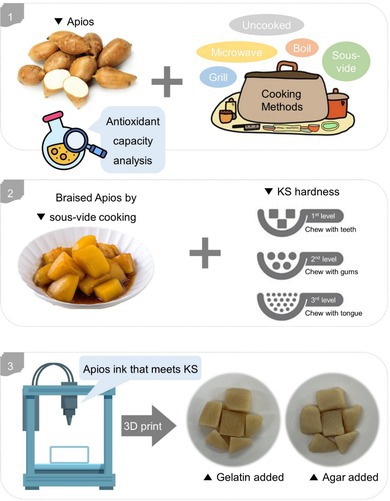

## 1 Introduction

As people age, their physical functions generally decline, particularly among the elderly people, whose chewing ability significantly diminishes. This reduction in chewing capacity makes it challenging to consume foods with hard or tough textures (Kim and Iida 2023). Consequently, this can lead to a significant decline in quality of life, as older adults become more susceptible to nutritional deficiencies. Therefore, it is crucial to enhance the elderly people's access to nutritious food. For instance, root vegetables have gained attention as an excellent source of nutrition, containing a variety of physiologically active compounds and being rich in minerals and vitamins. These foods are recommended for the elderly people to help prevent cardiovascular diseases, dementia, osteoporosis, cancer, and other health issues. This study aimed to develop elder‐friendly food that meets the hardness standards set by Korean industrial standards (KS) by utilizing Apios ( *Apios americana* Medicus).

Apios is a tuber‐bearing leguminous plant native to eastern North America (Li et al. 2021). The tuber of Apios was considered a staple food for over 100 years by North American Indigenous people and early European settlers, and it is now cultivated in North America, East Asia, Europe, and other regions. Apios has been reported to exhibit strong resistance to various adverse environmental conditions and can be cultivated at low economic costs. Furthermore, several studies have demonstrated that Apios is rich in essential nutrients and microelements, including starch, protein, lipids, flavonoids, phenolics, isoflavones, and polysaccharides. Given these attributes, Apios, which has a potato‐like taste and high nutritional value, appears to be an excellent food ingredient for the elderly people, which can help address nutritional imbalances. However, Apios contains solanine, so it is advisable to cook and consume it, particularly for children and the elderly people. The nutritional properties, such as antioxidant capacity, can vary depending on the cooking method. Therefore, this study aimed to investigate the changes in antioxidant activity associated with different cooking methods of Apios.

Recently, Apios has been cultivated in regions such as Gyeongsangnam‐do and Chungcheongbuk‐do in Korea; however, achieving uniform size in cultivation remains challenging (Park and Kim 2020). Additionally, smaller varieties of Apios, aside from the commercially available ones, possess a limitation: They are primarily used as bulbs due to their excessive and coarse fibers, which are visible to the naked eye. Many root vegetables, including Apios, tend to be tough because they are high in fiber and have a hard texture, making them difficult for the elderly people, who may have weakened chewing abilities, to consume. Recent research has increasingly focused on finding ways to facilitate the consumption of root vegetables to help the elderly people maintain balanced nutrition across various fields. However, not only is taste important but also the visual appeal of food also plays a significant role in the pleasure of eating. Unfortunately, research on the development of elder‐friendly foods that consider these gastronomic characteristics is still insufficient. For instance, most elder‐friendly foods available on the market are liquid forms, such as porridge or mousse, which are typically round or square. This study aimed to develop elder‐friendly foods that take gastronomic characteristics into account by utilizing 3D printing technology. The goal is to restore the original appearance of Apios using a 3D food printer, rather than relying on simple shapes like circles or squares. By creating 3D‐printed elder‐friendly foods that adjust rheological properties for easier chewing, we hope to meet the diverse demands of elderly consumers. Additionally, this initiative aims to support the growth of the elder‐friendly food industry by leveraging domestic agricultural resources.

## 2 Materials and Methods

### 2.1 Material

The Apios utilized in this research was collected in Jinju‐si, Gyeongsangnam‐do, South Korea, in February 2024. It was peeled and divided into 100 g portions, prepared in the following ways: (1) raw Apios (uncooked), (2) grilled in an oven at 150°C for 25 min, (3) boiled in 1 L of water at 100°C for 10 min, (4) microwaved at 700 W for 2 min, and (5) sous‐vide cooked in water at 80°C for 40 min.

### 2.2 Antioxidant Capacity

The prepared Apios, which was grilled, boiled, microwaved, and sous‐vide, underwent freeze‐drying at −70°C for 72 h before being ground into a powder. This powdered Apios was then combined with 70% ethanol at a 1: 10 (w/v) ratio and agitated for 24 h in an incubator set at 27°C. The resulting mixture was filtered using Whatman filter paper (Washington, DC, USA) and utilized as a sample for tests measuring antioxidant capacity. The following contents from Sections 2.2.1 to 2.2.5 were assessed using the procedure outlined by Kim and Iida (2023).

#### 2.2.1 Flavonoid Content

To prepare the mixture, 2 mL of the sample was combined with 2 mL of 10% AlCl_3_∙6H_2_O, 2 mL of 1 M CH_3_COOK, and 11.2 mL of distilled water. This mixture was allowed to sit in the dark for 30 min. The absorbance of the resulting solution was then measured at 415 nm, using quercetin as the standard solution.

#### 2.2.2 Phenolic Content

Initially, 0.5 mL of the sample was mixed with 2.5 mL of 2 N Folin–ciocalteu's phenol reagent (Sigma‐Aldrich, St. Louis, MO, USA), followed by the addition of 7.5 mL of 20% Na_2_CO_3_. Distilled water was then added to bring the total volume to 50 mL. The mixture was allowed to sit in the dark for 2 h, after which the absorbance was measured at 765 nm, with gallic acid used as the standard solution.

#### 2.2.3 Ferric Reducing Antioxidant Power

A mixture was prepared by combining 1 mL of the sample with 2.5 mL of 0.2 M sodium phosphate buffer (pH 6.6). Subsequently, 2.5 mL of 1% K_3_Fe(CN)_6_ was added, and the mixture was incubated in a water bath at 50°C for 20 min. Following this, 2.5 mL of 10% C_2_HCl_3_O_2_ was added to the solution, and a 2.5 mL of supernatant was obtained by centrifugation at 3000 rpm for 10 min. To this supernatant, 2.5 mL of distilled water and 0.5 mL of 0.1% FeCl_3_ were added, and the absorbance was measured at 700 nm, with ascorbic acid serving as the standard solution.

#### 2.2.4 DPPH Free Radical Scavenging Activity

An experimental group was prepared by mixing 5 mL of a sample, 1 mL of a 55 μM 1,1‐diphenyl‐2‐picrylhydrazyl (DPPH), and 6 mL of 99.99% ethanol. This mixture was allowed to stand in the dark for 30 min before measuring the absorbance at 517 nm. A control group was prepared by mixing 6 mL of a 55 μM DPPH solution with 6 mL of 99.99% ethanol, and the experiment was conducted in the same manner as the experimental group. The measured absorbance was calculated using the following equation: DPPH free radical scavenging activity (%) = {(Absorbance_control − Absorbance_experimental)/Absorbance_control} × 100.

#### 2.2.5 ABTS Free Radical Scavenging Activity

ABTS stock solution was prepared by mixing 1 mL of 7 mM 2,2‐azino‐bis(3‐ethylbenzenothiazoline)‐6 sulfonic acid (ABTS) with 1 mL of 2.45 mM K_2_S_2_O_8_, and then allowing it to sit in the dark for 24 h. To prepare the ABTS working solution, 70% ethanol was added to the ABTS stock solution until the absorbance measured at 734 nm reached 1.00 ± 0.02. For the experimental group, 2 mL of the ABTS working solution was mixed with 0.2 mL of the sample, and the absorbance was measured at 734 nm. A control group was prepared by mixing 0.2 mL of 70% ethanol in place of the sample, and the experiment was conducted in the same manner as the experimental group. The measured absorbance was calculated using the following equation: ABTS free radical scavenging activity (%) = {1 − (Absorbance_experimental/Absorbance_control)} × 100.

### 2.3 Development of Elder‐Friendly Food With Apios Cooked With Optimized Cooking Method

#### 2.3.1 Manufacturing Method of Mousse Type Sous‐Vide Apios

100 g of Apios, 2 teaspoons of soy sauce, and 2 teaspoons of oligosaccharide were placed in a polyvinyl bag for sous‐vide cooking. The bag was sealed and then cooked sous‐vide at 80°C for 40 min. After cooking, water was added to achieve 20% of the total weight. The mixture was rapidly frozen in a deep freezer at −40°C for 24 h and then ground using a microgrinder, specifically a Pacojet (Pacojet 2 Plus, Pacojet International AG, Rotkreuz, Switzerland). Gelatin or agar was incorporated in varying ratios of 2%, 4%, 6%, 8%, 10%, 12%, and 14%, respectively. The mixture was then refrigerated at 4°C for 1 h before being used as a sample for texture analysis.

#### 2.3.2 Texture Property

The hardness of the sample was measured using a texture analyzer (TA.XTExpressC, Stable Micro Systems, Surrey, UK). The measurement conditions were set as follows: pretest speed 3.0 mm/s, test speed 3.0 mm/s, posttest speed 3.0 mm/s, test distance 2.0 mm, time 3.00 s, and trigger force 0.0098 N.

### 2.4 Preparation for 3D Printed Elder‐Friendly Sous‐Vided Apios

Using the regression equation derived from the texture experiment above, the 3D printer ink was prepared according to the midpoint of the second level of KS (35,000 N/m^2^) as follows: (1) 30 g of braised Apios, 6.3 g of gelatin, and 6 g of water; (2) 30 g of braised Apios, 3.9 g of agar, and 6 g of water.

#### 2.4.1 3D Printing Design

**FIGURE 1:**
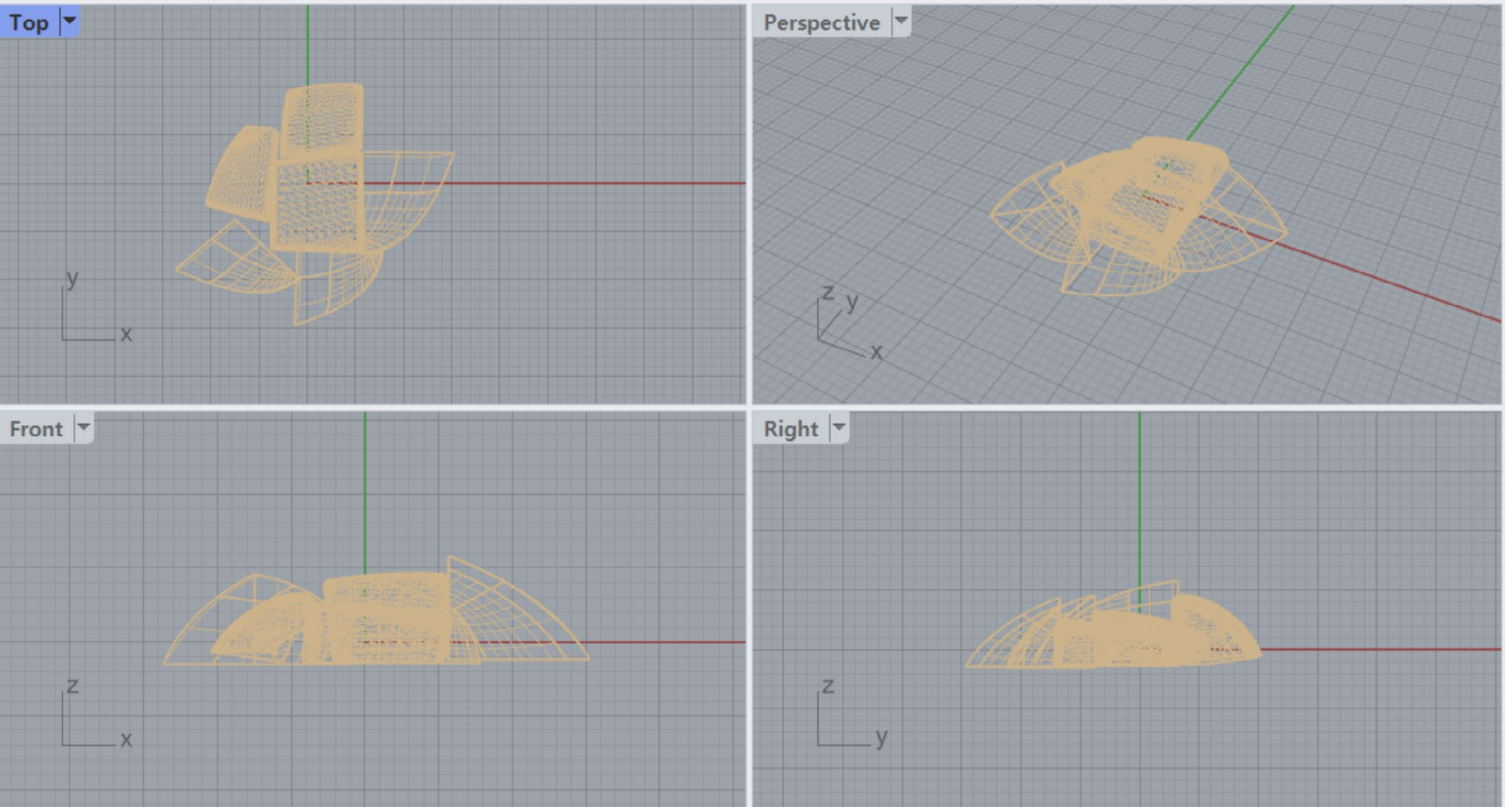
Braised Apios shape modeled by Rhino 7.

After modeling the shape of the braised Apios using Rhino 7 (Rhinoceros 7, TLM Inc., Seattle, WA, USA) (Figure 1), the model was sliced with Cura (Ultimaker, Utrecht, Netherlands) for printing. Slicing is the process of dividing a 3D model created in Rhino 7 into very thin layers and generating a command, commonly referred to as g‐code, which instructs the 3D printer on where and how much material to deposit in each layer as the printer's nozzle moves. The output dimensions were set to 150 mm along the *X*‐axis, 150 mm along the *Y*‐axis, and 150 mm along the *Z*‐axis, utilizing a 3D printer (3dcook‐c1, 3DCOOK, Degu, Korea).

#### 2.4.2 Sensory Property

Sensory evaluation was conducted on 20 professional panels specializing in food nutrition (SMWU‐2404‐HR‐013). The samples included two 3D‐printed, elder‐friendly braised Apios, one with added gelatin and the other with agar. The panels assessed the samples based on taste, flavor, appearance, texture, and overall preference using a 7‐point scale.

### 2.5 Statistical Analysis

Statistical analysis was conducted using the SPSS program. A one‐way analysis of variance (ANOVA) was employed to compare the average differences among multiple groups, specifically examining antioxidant activity based on the recipe and hardness in relation to the type and concentration of the gelling agent. This analysis aimed to determine whether the average differences were statistically significant. If the results were significant at *p* < 0.05, Duncan's post hoc test was applied. Additionally, a *t*‐test was performed to assess the differences between two independent groups concerning sensory properties based on the type of gelling agent.

## 3 Results and Discussion

### 3.1 Antioxidant Capacity

#### 3.1.1 Flavonoid Content

The flavonoid content of raw Apios was measured at 14.879 mg QE/g (Table 1), which is lower than that of sweet potato (29.78 mg/g; Kim et al. 2019) but slightly higher than that of yam (10.94 mg/g; Li et al. 2018) and comparable to that of potato (13.10 mg/g; Park et al. 2014). Flavonoids are natural compounds found in plants, including fruits and vegetables, and are associated with various health benefits, such as reduced inflammation. A previous study indicated that elderly individuals who consumed fewer flavonoid‐rich foods were two to four times more likely to develop Alzheimer's disease and dementia over a 20‐year period compared to those who consumed adequate amounts of these foods (Shishtar et al. 2020). Given these considerations, Apios may be as valuable as potato and yam, which are root vegetables with high consumption rates.

**TABLE 1:** Antioxidant capacity of Apios by cooking method.

	Flavonoid content (mg QE/g)	Phenolic content (mg GAE/g)	FRAP (mg ascorbic acid/g)	DPPH free radical scavenging activity (%)	ABTS free radical scavenging activity (%)
	Mean ± SD				
Raw	14.879 ± 0.026^a^	44.657 ± 0.000^a^	68.365 ± 0.137^a^	39.322 ± 0.028^a^	66.861 ± 0.378^a^
Grilled	12.591 ± 0.481^c^	39.260 ± 0.740^c^	54.635 ± 1.755^d^	34.708 ± 1.569^d^	60.848 ± 0.728^b^
Boiled	11.667 ± 0.353^d^	36.562 ± 1.098^d^	53.206 ± 2.612^d^	35.525 ± 1.463^cd^	56.345 ± 1.361^c^
Microwaved	13.788 ± 0.486^b^	42.213 ± 0.764^b^	58.365 ± 1.689^c^	37.281 ± 0.411^bc^	61.261 ± 0.713^b^
Sous‐vided	13.970 ± 0.486^b^	43.038 ± 0.909^b^	63.841 ± 2.263^b^	38.547 ± 0.774^ab^	65.602 ± 1.119^a^
*F*‐value (*p*)	28.560*** (0.000)	28.560*** (0.000)	49.348*** (0.000)	10.658** (0.001)	58.185*** (0.000)

*Note:* Different letters in the same column (a–d) indicate a significant difference.

Abbreviation: SD, standard deviation.

** *p* < 0.01.

*** *p* < 0.001.

On the other hand, Apios contains solanine, so it is advisable for the elderly people to cook it. In this study, various cooking methods were employed, and the flavonoid content of each method was measured. The results indicated that the flavonoid content of sous‐vide Apios (13.970 mg QE/g) was significantly higher than that of the other methods. The flavonoid content ranked in the following order: microwave cooking, baking, and boiling. Generally, dietary flavonoids are susceptible to thermal processing due to their lower stability, which often impairs their bioactivity after exposure to heat (Gao et al. 2022). Previous studies have shown that grilling and boiling also reduce the flavonoid content in eggplant, highlighting that the thermal stability of dietary flavonoids is influenced by various factors, including cooking techniques, heating temperature, heating duration, and food matrices. While dietary flavonoids are sensitive to thermal conditions, some research suggests that thermal processing can enhance their biological activity. Therefore, based on the results of this study, the intake of sous‐vide Apios is recommended.

#### 3.1.2 Phenolic Content

The phenolic content of raw Apios was measured at 44.657 mg GAE/g (Table 1), which is lower than that of sweet potato (55.26–69.47 mg/g; Im and Suh 2009), but slightly higher than that of yam (38.13 mg/g; Li et al. 2018), and comparable to that of potato (48.20 mg/g; Park et al. 2014). Phenols are secondary metabolites that are widely distributed in the plant kingdom and are known to contribute to health maintenance and disease prevention due to their physiological functions, which include antimicrobial, anticancer, antithrombotic, and antioxidant effects (Im and Suh 2009). Recently, as human lifespans have increased and the desire for health maintenance has grown, there has been a notable rise in antioxidant‐rich foods that can help suppress aging. In line with this trend, this study aimed to explore the potential of Apios as a valuable food resource.

As a result of various cooking methods, sous‐vide cooking produced the highest phenolic content in Apios. This was followed in descending order by microwave cooking, baking, and boiling. According to a previous study, the elution of phenols increases when the glycosidic bonds of polyphenol sugars are broken, which may occur as the microstructure of vegetables changes due to various cooking factors (Ko et al. 2016). However, in sous‐vide cooking, it has been reported that the elution of water‐soluble components is minimized because the food does not come into direct contact with water, leading to less nutrient loss and potentially increased nutrient content. Conversely, with microwave cooking, prolonged exposure to electromagnetic waves can damage cell tissue, resulting in a loss of phenolic compounds. Therefore, based on these findings, the intake of sous‐vide Apios is recommended.

#### 3.1.3 Ferric Reducing Antioxidant Power

The FRAP of raw Apios was measured at 68.365 mg of ascorbic acid/g (Table 1). This value was lower than that of radish (90.0 mg/g) and turmeric (169.1 mg/g), but higher than that of carrot (13.2 mg/g) and red beet (43.3 mg/g; Tiveron et al. 2012). When comparing cooking methods, the antioxidant capacity of sous‐vide Apios was significantly the highest, followed by microwave cooking, baking, and boiling. Previous studies have shown that when comparing conventional cooking methods with sous‐vide, 11 vegetables (including red onion and beetroot) exhibited higher antioxidative potential after being cooked using the sous‐vide method than after conventional cooking.

However, based on the results of these studies, it cannot be definitively concluded that the sous‐vide process has a beneficial effect on increasing antioxidative potential. Nevertheless, an increase in antioxidative potential was observed in certain vegetables. This effect may be attributed to the destruction of vegetable cells during traditional cooking, which leads to the leaching of valuable nutrients into the cooking water. In contrast, the sous‐vide method, which employs low temperatures and vacuum packaging, can help preserve the cellular structure of vegetables and retain components with antioxidative properties, including vitamins and minerals, among other nutrients.

#### 3.1.4 DPPH Free Radical Scavenging Activity

The DPPH free radical scavenging activity of raw Apios was 39.322% (Table 1), which is lower than that of yam (74.57%; Li et al. 2018) but higher than that of potato (16.13%; Bembem and Sadana 2013). It is also comparable to sweet potato (41.20%; Lee et al. 2007) and cassava (35.18%; Kim and Iida 2023). Free radicals are unstable atoms that can damage cells, leading to illness and aging. They are generated as a result of impaired or disrupted mitochondrial respiratory processes (Kim and Iida 2023). Free radical scavengers are compounds that neutralize these harmful free radicals. As the elderly population increases, there is growing interest in foods with strong antioxidant properties, such as those exhibiting DPPH free radical scavenging activity. According to the results of this study, Apios possesses antioxidant properties comparable to those of potatoes and sweet potatoes, which are widely consumed root vegetables. Therefore, it is considered a valuable ingredient for elder‐friendly foods aimed at preventing aging.

As a result of exploring various cooking methods to utilize Apios, the DPPH free radical scavenging activity of sous‐vide Apios was significantly the highest. Following this, the antioxidant activity was notably high in the order of microwave cooking, boiling, and baking. In a previous study vacuum cooking at low temperatures demonstrated several advantages, with 14 out of 22 tested vegetables (including beetroot, carrot, parsley root, and kohlrabi) showing a significant increase in antioxidant activity. For cooking methods such as sous‐vide, it has been reported that since the food does not come into direct contact with water, the leaching of water‐soluble components is minimized, resulting in minimal nutrient loss or even an increase in nutrient content. Therefore, based on these findings, the intake of sous‐vide Apios is recommended.

#### 3.1.5 ABTS Free Radical Scavenging Activity

The ABTS free radical scavenging activity of raw Apios was 66.861% (Table 1), which is lower than that of turmeric (118.6%) but higher than that of red beet (2.5%) and turnip (6.5%), and comparable to that of radish (61.7%; Tiveron et al. 2012). Previous studies indicate that cooking methods such as baking or boiling tend to damage vegetable cells due to high temperatures and moisture. In contrast, sous‐vide cooking minimizes direct contact with water, reducing the leaching of water‐soluble components and resulting in less nutrient loss or even an increase in nutrient content. Considering these findings, Apios demonstrates significant antioxidant activity, making it a valuable ingredient for elder‐friendly foods aimed at preventing aging. Therefore, sous‐vide is recommended as the preferred cooking method.

### 3.2 Texture of Mousse‐Type Braised Apios

**FIGURE 2:**
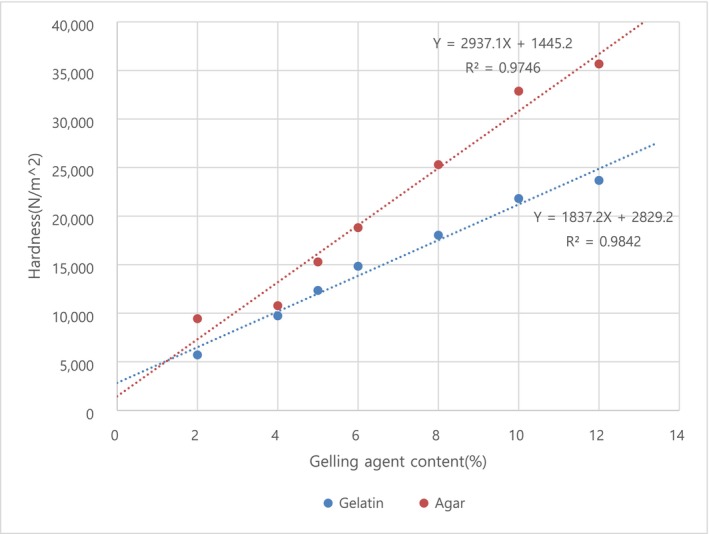
Regression equation for hardness of mousse‐type braised Apios according to gelling agent. The *Y*‐scale means hardness (N/m^2^), and the *X*‐scale means gelling agent content (%). The coefficient of determination (*R* ^2^) is a number between 0 and 1 that measures how well the statistical model predicted the outcome, indicating that the higher this is, the better suited it is to the observations. The first level of hardness of KS is 55,000–500,000 N/m^2^, the second level is 22,000–50,000 N/m^2^, and the third level is less than 20,000 N/m^2^.

As the ratio of gelatin added increased, the hardness of the mousse‐type braised Apios significantly increased (Table 2). The derived regression equation for this relationship is *Y* = 1837.2*X* + 2829.2 (Figure 2). Based on the derived regression equation, the appropriate gelatin addition ratios for each level of chewability (KS) are as follows: For hardness that can be chewed with teeth (Level 1), it is 28.397% or more; for hardness that can be chewed with gums (Level 2), it is between 10.435% or more and 25.675% or less; and for hardness that can be chewed with the tongue (Level 3), it is 9.346% or less. Similarly, as the ratio of agar added increased, the hardness of the mousse‐type braised Apios also significantly increased (Table 2). The regression equation for this relationship is *Y* = 2937.1*X* + 1445.2. Based on this equation, the appropriate agar addition ratios for each level of chewability (KS) are as follows: for hardness that can be chewed with teeth (Level 1), it is 18.234% or more; for hardness that can be chewed with gums (Level 2), it is between 6.998% or more and 16.532% or less; and for hardness that can be chewed with the tongue (Level 3), it is 6.317% or less.

**TABLE 2:** Hardness (N/m^2^) of mousse‐type braised Apios according to gelling agent content.

	Gelatin	Agar	*t*‐value (*p*)
	Mean ± SD		
2%	5709.300 ± 183.800^g^	9440.700 ± 444.700^c^	−13.430** (0.002)
4%	9732.300 ± 348.800^f^	10,781.000 ± 64.600^c^	−5.120* (0.031)
6%	12,348.000 ± 317.700^e^	15,285.700 ± 178.800^c^	−13.957** (0.001)
8%	14,840.300 ± 33.600^d^	18,814.300 ± 752.100^c^	0.321 (0.765)
10%	18,036.300 ± 495.900^c^	25,297.700 ± 2532.800^b^	−5.209** (0.006)
12%	21,810.700 ± 730.300^b^	32,871.000 ± 1100.000^ab^	−13.313*** (0.000)
14%	23,677.300 ± 1033.000^a^	35,672.000 ± 1992.300^a^	−9.258** (0.003)
*F*‐value (*p*)	418.952*** (0.000)	22.915*** (0.000)	

*Note:* Different letters in the same column (a–g) indicate a significant difference.

Abbreviation: SD, standard deviation.

* *p* < 0.05.

** *p* < 0.01.

*** *p* < 0.001.

Meanwhile, agar has a greater influence on hardness than gelatin due to several key differences between the two, including their sources, compositions, forms, and physical properties (Igarashi et al. 2002). First, agar and gelatin exhibit distinct health‐promoting properties. Agar is a jelly‐like substance derived from red seaweeds and is classified as a plant‐based hydrocolloid. It is rich in dietary fiber, which offers numerous health benefits, such as enhancing digestive function and preventing constipation. Due to its limited digestibility, agar promotes a prolonged feeling of satiety and helps stabilize blood glucose levels, preventing rapid fluctuations. Additionally, agar is a source of various essential minerals, including calcium, potassium, magnesium, iron, and vitamins B, E, and K. In contrast, gelatin is a translucent, colorless, and flavorless natural protein typically obtained from collagen extracted from animal tissues.

Gelatin is rich in collagen, a protein that is an essential component of our skin and joints. Previous studies indicate that the consumption of gelatin, particularly when combined with vitamin C, enhances the condition of the musculoskeletal system, which is especially beneficial for patients with joint diseases (Shaw et al. 2017). Additionally, gelatin has a positive effect on wound healing, promotes youthful skin, and, like agar, helps reduce feelings of hunger, thereby supporting weight loss. Given these benefits, gelatin may serve as a nutritionally advantageous gelling agent in the production of elder‐friendly foods.

Agar and gelatin possess distinct chemical properties, resulting in variations in texture and taste (Igarashi et al. 2002). Gelatin, when used in low ratios, produces a soft gel, while higher ratios yield a stretchy, chewy, bouncy, and elastic gel. Additionally, gelatin melts at body temperature and has a low melting point, allowing for a more pronounced flavor experience. In contrast, agar consistently forms a brittle gel. Due to its high melting point, agar does not dissolve in the mouth, which means that its pieces remain perceptible when consumed. Furthermore, while gelatin is generally flavorless, agar has a subtle seaweed taste that may be detectable in certain recipes. These differences in mouthfeel and flavor are significant to consumers and can influence their purchasing decisions.

When developing elder‐friendly foods, it is essential to consider dietary restrictions, temperature sensitivity, and ingredient ratios to achieve the desired texture and taste during the manufacturing process. Taking all of these factors into account, it is crucial to also consider the dietary preferences of the elderly people and the potential health benefits that various gelling agents, includingagar and gelatin, may offer. This study aimed to facilitate adjustments based on personal preferences in the production of elder‐friendly foods. Furthermore, the findings of this study are expected to serve as foundational data to support the industrialization of elder‐friendly food products.

### 3.3 3D Printed Elder‐Friendly Braised Apios

#### 3.3.1 Optimized 3D Printing Condition

**FIGURE 3:**
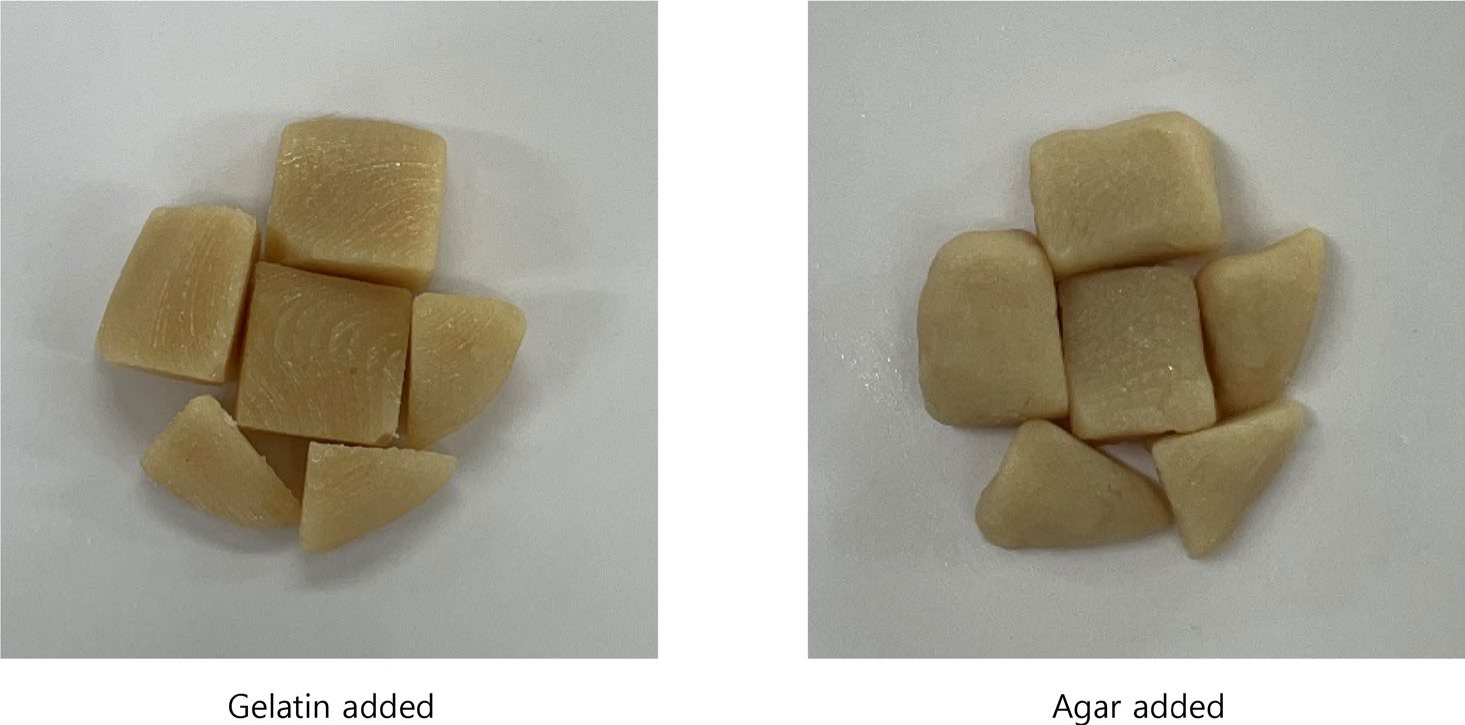
3D‐printed elder‐friendly braised Apios.

Table 3 presents the conditions under which the 3D printer ink was output stably. Among the output conditions for food ink containing gelatin and food ink containing agar, the print speed and initial layer speed varied. These two factors significantly influence the output, as they are closely related to the ink's viscosity. Generally, high‐viscosity ink allows for a higher output speed because it flows more readily than low‐viscosity ink. Consequently, in this study, the speed of the ink with added gelatin was greater than that of the ink with added agar. Establishing optimal output conditions for each 3D printer's food ink is crucial, as variations in the quality of the final print can arise when samples with differing rheological properties are printed under identical conditions (Kim et al. 2024). Therefore, this study aimed to develop visually appealing, easy‐to‐chew foods with enhanced nutritional value using gelling agents (gelatin and agar) and a 3D printer, with the results illustrated in Figure 3.

**TABLE 3:** Optimized output conditions of elder‐friendly braised Apios ink for 3D printer.

	Agar added ink	Gelatin added ink
Cylinder	50 mL syringe‐shaped cylinder Stainless steel	50 mL syringe‐shaped cylinder Stainless steel
Scale	*X*: 34 mm *Y*: 30 mm *Z*: 8.8 mm	*X*: 34 mm *Y*: 30 mm *Z*: 8.8 mm
Quality	Layer height: 0.3 mm Initial layer height: 0.3 mm Line width: 1.0 mm	Layer height: 0.3 mm Initial layer height: 0.3 mm Line width: 1.0 mm
Walls	Wall thickness: 3.0 mm Wall line count: 3	Wall thickness: 3.0 mm Wall line count: 3
Top/Bottom	Top/Bottom thickness: 0.8 mm Top/Bottom layers: 3	Top/Bottom thickness: 0.8 mm Top/Bottom layers: 3
Infill	Infill density: 20.0% Infill pattern: Zig Zag Infill overlap: 0.3 mm	Infill density: 20.0% Infill pattern: Zig Zag Infill overlap: 0.3 mm
Material	Printing temperature: 30.0°C Build plate temperature: 30.0°C	Printing temperature: 30.0°C Build plate temperature: 30.0°C
Speed	Print speed (Infill, Wall, Top, Bottom): 20.0 mm/s Travel speed: 50.0 mm/s Initial layer speed: 20.0 mm/s	Print speed (Infill, Wall, Top, Bottom): 35.0 mm/s Travel speed: 50.0 mm/s Initial layer speed: 30.0 mm/s
Travel	Enable retraction Retraction distance: 6.5 mm Retraction speed: 25.0 mm/s Retraction minimum move: 2.0 mm	Enable retraction Retraction distance: 6.5 mm Retraction speed: 25.0 mm/s Retraction minimum move: 2.0 mm
Cooling	Enable print cooling Fan speed: 100.0%	Enable print cooling Fan speed: 100.0%
Support	Generate support Support pattern: Zig Zag Support density: 15.0% Support Z distance: 0.1 mm X/Y support distance: 0.7 mm	Generate support Support pattern: Zig Zag Support density: 15.0% Support Z distance: 0.1 mm X/Y support distance: 0.7 mm
Build plate adhesion	Build plate adhesion type: Skirt Skirt line: 3 Skirt distance: 5.0 mm	Build plate adhesion type: Skirt Skirt line: 3 Skirt distance: 5.0 mm

It has been confirmed that both agar and gelatin can be utilized in the production of 3D‐printed elder‐friendly foods. Additionally, various types of gelling agents can be employed to achieve specific objectives, such as catering to consumer preferences, enhancing texture, and adjusting hardness. This finding indicates that 3D printers can successfully replicate the visual appearance of elder‐friendly foods. Consequently, the use of 3D printing technology may lead to high‐quality, easy‐to‐chew foods for the elderly people. This innovation is significant, as traditional dysphagia diets are typically limited to porridge or puree. In contrast, 3D‐printed elder‐friendly foods closely resemble their original counterparts, likely improving the appetite of elderly individuals.

#### 3.3.2 Sensory Property

All preferences were higher for gelatin than for agar when added to 3D‐printed elder‐friendly braised Apios; however, there was no significant difference in preference for taste and flavor (Table 4). While gelatin is flavorless, agar has a subtle seaweed taste that may be noticeable in certain foods (Igarashi et al. 2002). Therefore, considering these differences, gelling agents should be used appropriately in food preparation. In this study, neither gelling agent significantly affected the taste and flavor of elder‐friendly braised Apios, indicating that both gelatin and agar can be utilized in the development of mousse‐type elder‐friendly foods. On the other hand, there was a significant difference in texture and appearance preferences between the gelatin addition group and the agar addition group. Gelatin has a softer and more elastic texture than agar, which has a firmer consistency (Igarashi et al. 2002). This difference arises from the distinct structures formed by each gelling agent. When using gelatin, the resulting food tends to be more jelly‐like, while agar produces a dessert with a solid form that lacks the springiness of gelatin when touched. Considering these factors, it is essential to select an appropriate gelling agent based on the desired texture. This approach will facilitate the development of a mousse‐type food that is easy to chew and caters to various consumer preferences, particularly for elder‐friendly options.

**TABLE 4:** Sensory property of 3D‐printed elder‐friendly braised Apios.

	Gelatin	Agar	*t*‐value (*p*)
	Mean ± SD		
Taste preference	4.167 ± 1.472	3.500 ± 1.761	0.712 (0.493)
Flavor preference	4.500 ± 1.643	3.667 ± 1.211	1.000 (0.341)
Appearance preference	6.000 ± 1.265	2.333 ± 0.816	5.966*** (0.000)
Texture preference	5.167 ± 1.472	3.000 ± 0.632	3.313** (0.008)
Overall preference	5.000 ± 1.265	3.167 ± 1.169	2.607* (0.026)

Abbreviation: SD, standard deviation.

* *p* < 0.05.

** *p* < 0.01.

*** *p* < 0.001.

## 4 Conclusion

In this study, we investigated the antioxidant capacity of Apios by comparing various cooking methods and their potential applicability in elder‐friendly foods. The results demonstrated that the antioxidant properties of Apios were significantly influenced by the preparation methods. Specifically, sous‐vide cooking was found to retain higher levels of antioxidants than that of other methods, suggesting that this technique is preferable for maximizing the health benefits of Apios, particularly for the elderly. Additionally, textural analysis indicated that the mousse‐type braised Apios could be effectively incorporated into soft‐textured elder‐friendly foods, ensuring both nutritional value and ease of consumption for the elderly people. Furthermore, the development of 3D‐printed elder‐friendly Apios shows significant potential for enhancing the nutritional quality and accessibility of food for the elderly.

By leveraging 3D printing technology, it is possible to customize the texture, shape, and nutrient composition of Apios‐based food products to meet the specific dietary needs and swallowing capabilities of seniors. Given these factors, it is anticipated that the high antioxidant capacity of Apios, combined with its adaptability in 3D printing, positions it as a promising ingredient for personalized, health‐promoting meals for the elderly people. Further research is needed to explore the scalability of this approach and its clinical health benefits. Additionally, studies involving food materials other than Apios may yield significantly different results. In other words, since research outcomes can vary considerably depending on the food material used, further studies that account for this variability should be conducted.

## Author Contributions

**Dah‐Sol Kim:** conceptualization (equal), data curation (equal), formal analysis (equal), investigation (equal), methodology (equal), resources (equal), software (equal), visualization (equal), writing – original draft (equal), writing – review and editing (equal). **Ju Hong Park:** conceptualization (equal), funding acquisition (equal), investigation (equal), project administration (equal), supervision (equal), validation (equal), writing – review and editing (equal).

## Ethics Statement

The authors have nothing to report.

## Conflicts of Interest

The authors declare no conflicts of interest.

## Data Availability

The authors have nothing to report.
